# A disproportional rise in the growing submission rate to International Journal of Cardiology Heart & Vasculature during the COVID‑19 pandemic

**DOI:** 10.1016/j.ijcha.2021.100749

**Published:** 2021-02-27

**Authors:** Dominik Linz, Dobromir Dobrev

**Affiliations:** Department of Cardiology, Maastricht University Medical Centre and Cardiovascular Research Institute Maastricht, Maastricht, the Netherlands; Department of Cardiology, Radboud University Medical Centre, Nijmegen, the Netherlands; Centre for Heart Rhythm Disorders, University of Adelaide and Royal Adelaide Hospital, Adelaide, Australia; Department of Biomedical Sciences, Faculty of Health and Medical Sciences, University of Copenhagen, Copenhagen, Denmark; Institute of Pharmacology, West German Heart and Vascular Center, Faculty of Medicine, University Duisburg-Essen, Essen, Germany; Department of Medicine, Montreal Heart Institute and Université de Montréal, Montreal, Canada; Departments of Molecular Physiology & Biophysics, Baylor College of Medicine, Houston, USA

The COVID-19 pandemic has a significant impact on our daily life due to national lockdown restrictions and measures to reduce social contacts to prevent the wider spread of the virus. As one component of these restrictions, most cardiology conferences were canceled or turned into virtual online meetings [1] and in-person interactions were shifted to discussions on social media [2]. Furthermore, there were travel restrictions by universities and hospitals limiting the extent of business travels for physicians, physician scientists and basic scientists to a minimum. Herein, we report on a COVID-19 pandemic related transient rise in submission rate to the *International Journal of Cardiology Heart & Vasculature*, and discuss potential factors which may explain this observation.

Following the issued lockdown restriction initiated in mid-March 2020, a transient but substantial increase in submission rate of scientific manuscripts to this journal was noted (Fig. 1). Despite a consistent monthly increase in submissions from 2018 to 2020, the increase observed between May to August 2020 appears disproportional to be solely explained by the steadily growing interest to this Journal.

**Fig. 1:**
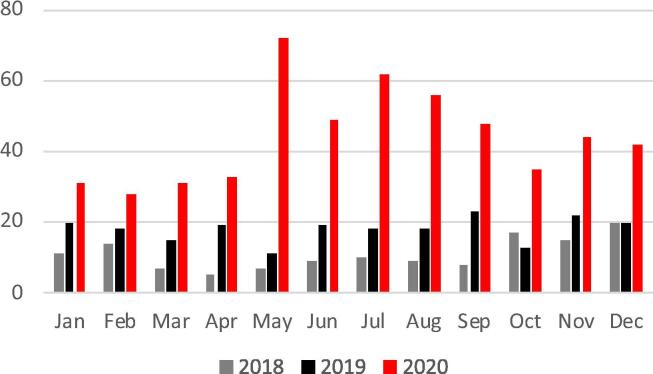
Submission rates of Y-axis: Number of manuscripts for the International Journal of Cardiology Heart & Vasculature 2018–2020.

Several factors might have contributed to this disproportional transient increase in submission rate. COVID-19 introduced a new research topic, which motivated several groups all over the world to investigate the putative interaction patterns with their respective research topic [3], [4], [5]. During the COVID-19 pandemic, a large proportion of elective cases in the hospitals all over the world have been postponed, which reduced the clinical workload in several specialties, including interventional cardiology, while the workload in the outpatient clinics increased [6]. Additionally, most in-person conferences were changed to hybrid or virtual online meetings, which potentially created spare time for research activities and preparation of manuscripts. However, for some clinicians and scientists, travelling time represents a protected and dedicated time to review articles and prepare manuscripts, which also dropped proportionally with the reduction in face-to-face meetings and conferences.

Although the lockdown restrictions during the COVID-19 pandemic were associated with a substantial increase in submission rate to the journal, it is unclear, whether the increase in submission rate will remain stable and ultimately translate into published manuscripts of better quality. Finally, we hope that this pandemic gets under control very quickly to allow face-to-face meetings and conferences, which created the basis of fruitful networking and productive collaborations for decades.

## Declarations

The authors report no relationships that could be construed as a conflict of interest.
